# Polygamy and safe sex negotiation among married women: evidence from Cameroon

**DOI:** 10.1186/s12879-023-08826-4

**Published:** 2023-11-22

**Authors:** Satveer Dhillon, Daniel Amoak, George N. Chidimbah Munthali, Yujiro Sano, Roger Antabe, Isaac Luginaah

**Affiliations:** 1 Department of Geography and Environment, Western University, 1151 Richmond St, London, ON N6A 5C2 Canada; 2 Mzuzu University, Mzuzu, Malawi; 3 Department of Sociology and Anthropology, Nipissing University, 100 College Dr, North Bay, ON P1B 8L7 Canada; 4 Department of Health and Society, University of Toronto Scarborough, 1265 Military Trail, Scarborough, ON M1C 1A4 Canada

**Keywords:** Polygamy, Safe sex negotiation, Marriage structure, Cameroon, Demographic and Health Survey

## Abstract

**Background:**

Research indicates that women in polygamous relationships may be exposed to unique sexual and reproductive health challenges. However, there are very few studies that examine whether polygamy is associated with safe sex negotiation among married women in sub-Saharan Africa, including Cameroon.

**Methods:**

Using the 2018 Cameroon Demographic and Health Survey, we apply logistic regression analysis to compare two indicators of safe sex negotiation (i.e., the ability to ask for condom use and refuse sex against their partner) between polygamous (*n* = 1,628) and monogamous (*n* = 5,686) women aged 15–49 years old.

**Results:**

We find that 67% and 50% of married women can ask for condom use and refuse sex against their partner, respectively. Multivariate analysis further reveals that women in polygamous relationships are less likely to report they can ask for condom use (OR = 0.71, *p* < 0.001) and refuse sex (OR = 0.64, *p* < 0.001) in comparison to their monogamous counterparts.

**Conclusions:**

Our analysis found that in Cameroon, women in polygamous relationships, Muslim women, married women with inadequate HIV knowledge, those who had never been tested for HIV and women with lower socioeconomic status are less likely to negotiate for safe sex. Based on these findings, we discuss several implications for policymakers, including the establishment of a comprehensive family planning educational program and the deployment of community health workers to disseminate educational initiatives pertaining to safe sex negotiation to community members.

## Introduction

Human immunodeficiency virus (HIV) remains a major public health challenge, especially in low-and middle-income countries. Sub-Saharan Africa (SSA) accounts for 71% of the global burden of HIV infection, despite only making up 12% of the global population [1]. Within this region, women and girls carry the highest HIV burden [2, 3]. In the context of Cameroon, for instance, despite the general decreasing trend in new HIV infections nationally, the prevalence rate remains higher among women (3.4%) than men (1.9%) [4, 5].

Importantly, a substantial number of new HIV infections occur among those in established relationships. While research has acknowledged that a complex set of factors may shape this gender disparity in the risk of HIV, married women’s ability to engage in safe sex has been identified as a critical determinant of HIV infections [6–8]. Safe sex negotiation, often measured with women’s ability to refuse sex or ask for condom use, is an important HIV prevention strategy that can decrease HIV transmission, especially when their partners are infected with HIV [9, 10].

Along with decreasing the risk of HIV transmission, safe sex negotiation can also decrease the risk of women being infected with other sexually transmitted infections (STIs), such as the Human Papilloma Virus (HPV), which has been observed to increase women’s HIV risk by 100% [11–13]. HPV is deemed a public health challenge, with the prevalence of HPV among the highest in SSA, compared to other regions in the world [14, 15]. Further, encouraging safe sex negotiation improves family planning, as studies have shown an inverse relationship between safe sex negotiation and high parity among women in SSA [16, 17]. Increasing a woman’s ability to negotiate for safe sex helps improve their participation in decision-making regarding family size and birth spacing [16, 18]. Additionally, unintended pregnancies and the associated health challenges can also be prevented if safe sex negotiation is encouraged among married women [17, 19].

However, research has reported within the context of SSA that married women may face unique sociocultural barriers when negotiating safe sex. For example, due to the cultural context of marriage in many SSA societies, men may have the final say in issues related to sex and reproduction in marriage, forcing women to disregard their own opinions and perspectives about safe sexual practices [7, 20–23]. Furthermore, some men may endorse cultural beliefs that men can enjoy more sexual freedom than women [10]. Moreso, in many African settings, sexual activities are perceived as essential expressions of a man’s masculinity, and men are praised for their sexual ability, including having multiple sexual partners [24]. Finally, in these settings, there is an emphasis on high fecundity within marriage, making it particularly challenging for women to justify refusing sex with their partners or asking for condom use [25]. These cultural dynamics of marriage may be particularly concerning, as research has shown that this can often enable men to assume unlimited sexual access to their wives [26]. In this context, married women may be highly exposed to the risk of HIV infections and other STIs within marital sexual relations when they engage in unprotected sex with their partners. Further, by negotiating for safe sex, a married woman can protect herself from unintended pregnancies and other health challenges [17]. To this end, safe sex negotiation can be an important strategy to improve several health and well-being outcomes in SSA among married women.

Reflecting on the importance of safe sex negotiation, previous studies have widely explored the factors associated with the ability to ask for condom use and refuse sex among married in SSA. For example, socioeconomic empowerment, measured with household wealth, education, and employment, has been associated with the ability to engage in safe sex negotiation [18, 27, 28]. Demographic characteristics such as religious affiliation, place of residence, and female genital mutilation or cutting (FGM/C) status have also been identified as determinants of safe sex negotiation in SSA [16, 27, 28]. Research has also shown that ability to ask for condom use and refuse sex is associated with psychosocial factors such as HIV/AIDS knowledge and the uptake of HIV testing [28, 29].

While these studies are relevant, only a few of them have sought to explore the association between household structure and safe sex negotiation in SSA, including Cameroon. The lack of research focus in this area is concerning, especially considering that SSA is one of the major regions in the world where polygamy is commonly practiced [30, 31]. Historically, polygamy was a fundamental part of family law in the majority of African countries [32], and to this date, it remains prevalent due to religious reasons and the desire to have several children that can provide farm labour, emotional support and help with older adult care [31, 33, 34]. However, women in polygamous unions have been observed to report lower levels of autonomy and may hesitate to question their husband’s decisions regarding safe sex, as they view themselves as easily substitutable, negatively impacting a number of health and well-being outcomes [30, 35, 36]. For example, studies have shown that polygyny escalates risky sexual behaviours, increasing the risk of HIV transmission [35, 37, 38]. Further, compounded with evidence of endemic gender inequality within Cameroon, it is imperative to understand the association between household structure and safe sex negotiation [39, 40].

To this end, the current study aims to address this void in the literature by exploring the association between household structure and two indicators of safe sex negotiation among married women in Cameroon—1) the ability to refuse sex and 2) the ability to ask for condom use. Using a nationally representative survey, the findings from this study will be useful for policymakers in Cameroon and beyond in the intersectoral context of gender inequality and HIV. Specifically, addressing this gap may be relevant for achieving Sustainable Development Goal (SDG) 5 (i.e., achieve gender equality and empower all women and girls) [41].

## Methods

We use the 2018 Cameroon Demographic and Health Survey (CDHS) to explore the prevalence and correlates of safe sex negotiation among married women in Cameroon. The 2018 CDHS is a nationally representative survey, which the National Institute of Statistics implemented in collaboration with the Ministry of Public Health. The CDHS utilized a multi-staged sampling framework in which systematic sampling with probability to size was applied to identify enumeration areas from which households were chosen. A total of 13,527 women aged 15–49 were interviewed, with a response rate of 98%. Due to the nature of the current study, we limited the sample to married women, leading to the final weighted sample size of 7,314 married women aged 15–49. More about this survey can be found elsewhere [42].

### Dependent variable

There are two dependent variables for this study. First, based on the question that asked respondents whether they can refuse sex with their partner, we constructed the dependent variable called ‘women’s ability to refuse sex’ (0 = no; 1 = yes). Second, respondents were further asked whether they can ask their partner for condom use during sex. Adopting this question, we constructed another dependent variable called ‘women’s ability to ask for condom use’ (0 = no; 1 = yes).

### Independent and control variables

The DHS measures the number of wives that the partner of a currently married woman has. This information is useful for documenting household structure, considering that monogamous marriage can be identified when women’s partners have no other wives; by contrast, those whose partners had more than one wife are classed as polygamous. This approach has been supported by previous studies [23, 43, 44], and we constructed this binary independent variable called ‘household structure’ (0 = monogamous; 1 = polygamous). In addition, the association between safe sex negotiation and household structure may be further impacted by other confounding factors. To account for possible confounders, we introduce a range of demographic (i.e., age, religion, region, and place), psychosocial (i.e., HIV knowledge and HIV testing), and socioeconomic factors (i.e., household wealth, education, and employment). Household wealth quintiles were constructed from a composite index based on the household ownership of consumer items, such as drinking water, car, and toilet facilities, among others. It is noteworthy that HIV knowledge was measured using two questions, indicating whether respondents know that (1) always using condoms during sex and (2) having only one sexual partner who had no other partner can reduce the risk of getting HIV. Respondents were coded ‘yes’ if they answer ‘yes’ to both questions and coded ‘no’ otherwise. In addition, household wealth quintiles were constructed from a composite index based on the household ownership of consumer items, such as drinking water, car, and toilet facilities, among others. Our decision to include these control variables has been supported by previous studies [16, 45, 46]. The summary of each variable has been shown in Table 1.

### Statistical analysis

We employed descriptive and regression analysis. First, we use descriptive statistics to understand the characteristics of the analytical sample. Second, we use logistic regression analysis to understand the associations between the dependent and independent variables. Models were built sequentially. There were four models in total—two each for the ability to refuse sex and ask for condom use. In Model 1 in Tables 3 and 4, we explored the unadjusted relationship between the dependent and independent variables, while control variables were further introduced in Model 2 in Tables 3 and 4. Results are shown with odds ratios (ORs). ORs larger than 1 indicate that women are more likely to have the ability to refuse sex and ask for condom use, while those smaller than 1 point to lower odds of having these abilities. All analyses used STATA 17 (State Corp, College Station, TX, USA). The ‘svy’ function is applied in statistical analysis to adjust for the cluster sampling design as well as sampling weights.

## Findings

Table 1 shows sample characteristics. About a third (33%) of women cannot refuse sex, while approximately half (50%) cannot ask for condom use. The majority of women are in a monogamous (78%) marriage. The largest age group is 25–29 (23%), followed by 30–34 (20%) and 20–24 (16%). We also find that the largest number of women are identified as Christian (68%), followed by Muslim (31%) and Traditionalist (2%). The majority of married women have a secondary level of education or lower (94%) and are employed (70%). In terms of locational characteristics, about half of respondents live in urban areas (48%), while the largest number of women live in Far-North (18%), followed by North-West (16%), West (10%), and Douala (10%). Meanwhile, 72% of married women have adequate HIV knowledge (meaning they are factually well-informed about HIV transmission), and nearly 80% have been tested for HIV.

**Table 1:** Sample characteristics

	Counts	Percentage
**Can refuse sex**		
No	2,413	32.99
Yes	4,901	67.01
**Can ask for condom use**		
No	3,647	49.86
Yes	3,667	50.14
**Household structure**		
Monogamous	5,686	77.74
Polygamous	1,628	22.26
**Region of residence**		
East	458	6.26
Adamawa	384	5.25
Centre	630	8.61
Douala	752	10.29
Far-North	1,362	18.63
Littoral	243	3.32
North	1,218	16.66
North-West	418	5.72
West	795	10.87
South	340	4.64
South-West	115	1.57
Yaoundé	599	8.18
**Place of residence**		
Urban	3,491	47.73
Rural	3,823	52.27
**Age of respondents**		
15–19	582	7.96
20–24	1,163	15.90
25–29	1,668	22.81
30–34	1,438	19.67
35–39	1,104	15.09
40–44	753	10.30
45–49	605	8.27
**Religion**		
Christian	4,736	64.75
Muslim	2,271	31.05
Traditionalist	152	2.08
None	130	1.78
Other	25	0.34
**Adequate HIV knowledge**		
Yes	5,261	71.93
No	2,053	28.07
**Ever been tested for HIV**		
Yes	5,830	79.71
No	1,484	20.29
**Education**		
Higher education	410	5.61
Secondary education	2,544	34.79
Primary education	2,253	30.80
No education	2,107	28.81
**Household wealth**		
Richest	1,395	19.07
Richer	1,409	19.26
Middle	1,450	19.82
Poorer	1,495	20.44
Poorest	1,566	21.41
**Employment**		
Employed	5,146	70.35
Unemployed	2,168	29.65
Total	7,314	100

Totals may not be exact due to rounding

Table 2 shows findings from the cross-classification analysis, revealing that there are significantly different characteristics between polygamous and monogamous women. For example, a smaller proportion of polygamous women reported that they are able to refuse sex (52% vs. 71%) and ask for condom use (25% vs. 57%) compared to monogamous women. In terms of residential region, the largest proportion of polygamous women lived in the North (34%), although their monogamous counterparts lived in Far-North (16%). There is also a sharp contrast for place of residence, revealing that monogamous women (71%) were more likely to live in rural areas than polygamous women (47%). In terms of religious affiliation, 73% of monogamous women were Christian, while 56% of polygamous women were Muslim. For psychosocial factors, it is interesting that polygamous women were less likely to have adequate HIV knowledge (67% vs. 73%) and HIV testing experience (67% vs. 83%), compared to their polygamous counterparts. In terms of socioeconomic characteristics, the proportion of those without formal education was larger among polygamous women (56%) compared to monogamous women (21%). Similarly, polygamous women (35%) were more likely to belong to the ‘poorest’ household compared to their monogamous counterparts (18%). As a subsequent analysis, we employed multivariate regression analysis to examine whether these demographic, psychosocial, and socioeconomic characteristics explain the differences in safe sex negotiation between polygamous and monogamous women

**Table 2:** Cross-classification analysis of selected variables by household structure

	Monogamous		Polygamous		*p*-value
	Counts	Percentage	Counts	Percentage	
**Can refuse sex**					***
No	1,629	28.65	784	48.14	
Yes	4,057	71.35	844	51.86	
**Can ask for condom use**					***
No	2,422	42.60	1,225	75.23	
Yes	3,264	57.40	403	24.77	
**Region of residence**					***
East	390	6.87	67	4.12	
Adamawa	246	4.33	138	8.47	
Centre	563	9.90	67	4.10	
Douala	709	12.47	43	2.66	
Far-North	934	16.43	428	2.63	
Littoral	227	3.99	16	0.99	
North	661	11.62	558	34.27	
North-West	383	6.73	36	2.19	
West	561	9.86	234	14.40	
South	319	5.61	21	1.26	
South-West	111	1.94	4	0.27	
Yaoundé	583	10.25	15	0.95	
**Place of residence**					***
Urban	3,027	53.23	464	28.52	
Rural	2,659	46.77	1,164	71.48	
**Age of respondents**					***
15–19	483	8.49	100	6.12	
20–24	975	17.14	189	11.58	
25–29	1,300	22.86	368	22.64	
30–34	1,125	19.78	314	19.28	
35–39	826	14.53	278	17.06	
40–44	550	9.67	203	12.47	
45–49	428	7.53	177	10.84	
**Religion**					***
Christian	4,156	73.09	580	35.62	
Muslim	1,357	23.87	914	56.13	
Traditionalist	77	1.35	76	4.64	
None	71	1.25	59	3.62	
Other	25	0.44	0	0.00	
**Adequate HIV knowledge**					***
Yes	4,164	73.22	1,097	67.41	
No	1,523	26.78	531	32.59	
**Ever been tested for HIV**					***
** Yes**	4,741	83.37	66.91	1,089	
No	946	16.63	33.09	539	
**Education**					***
Higher education	406	7.14	4	0.24	
Secondary education	2,325	40.89	219	13.45	
Primary education	1,767	31.08	485	29.81	
No education	1,188	20.88	920	56.49	
**Household wealth**					***
Richest	1,295	22.77	100	6.11	
Richer	1,187	20.88	222	13.63	
Middle	1,116	19.63	334	20.49	
Poorer	1,085	19.08	410	25.20	
Poorest	1,003	17.64	563	34.57	
**Employment**					n.s.
Employed	3,993	70.22	1,153	70.81	
Unemployed	1,693	29.78	475	29.19	
Total	5,686	100	1,628	100	

Totals may not be exact due to rounding; ***p < 0.001; n.s.=not significant; significance calculated based on x2 test

Table 3 shows findings from the logistic regression analysis, predicting factors associated with women’s ability to refuse sex. As shown in Model 1, we find at the bivariate level that polygamous women are less likely to be able to refuse sex than monogamous women (OR = 0.43, *p* < 0.001). This relationship remains significant after controlling for demographic, psychosocial, and socioeconomic factors in Model 2. Specifically, polygamous women are still less likely to be able to refuse sex than women in monogamous marriages (OR = 0.71, *p* < 0.001). In terms of the ability to ask for condom use, as shown in Model 1 of Table 4, we find at the bivariate level that polygamous women were less likely to be able to ask for condom use than monogamous women (OR = 0.24, *p* < 0.001). In Model 2, we observe that polygamous women are still less likely to be able to ask for condom use than monogamous women, even after accounting for demographic, psychosocial, and socioeconomic factors (OR = 0.71, *p* < 0.001).

**Table 3:** Logit models predicting whether married women can refuse sex

	Model 1			Model 2		
	OR	95% CI		OR	95% CI	
**Household structure**						
Monogamous	1.00			1.00		
Polygamous	0.43***	0.35	0.53	0.71***	0.59	0.86
**Region of residence**						
East				1.00		
Adamawa				0.68	0.45	1.03
Centre				0.64**	0.45	0.90
Douala				0.44**	0.26	0.75
Far-North				0.61*	0.40	0.95
Littoral				0.32***	0.21	0.49
North				0.29***	0.19	0.45
North-West				0.65	0.42	1.01
West				1.03	0.70	1.52
South				0.74	0.46	1.19
South-West				0.38***	0.23	0.62
Yaoundé				0.74	0.47	1.17
**Place of residence**						
Urban				1.00		
Rural				0.94	0.71	1.25
**Age of respondents**						
15–19				1.00		
20–24				0.96	0.74	1.25
25–29				1.19	0.93	1.53
30–34				1.28	0.98	1.68
35–39				1.39*	1.06	1.83
40–44				1.33	0.96	1.86
45–49				1.46*	1.04	2.06
**Religion**						
Christian				1.00		
Muslim				0.69***	0.56	0.86
Traditionalist				0.98	0.58	1.65
None				0.71	0.45	1.12
Other				1.53	0.53	4.42
**Adequate HIV knowledge**						
Yes				1.00		
No				0.58***	0.49	0.68
**Ever been tested for HIV**						
Yes				1.00		
No				0.72**	0.58	0.88
**Education**						
Higher education				1.00		
Secondary education				0.68	0.46	1.01
Primary education				0.49***	0.32	0.75
No education				0.38***	0.23	0.61
**Household wealth**						
Richest				1.00		
Richer				0.87	0.67	1.13
Middle				0.87	0.63	1.20
Poorer				0.71	0.50	1.02
Poorest				0.85	0.56	1.31
**Employment**						
Employed				1.00		
Unemployed				0.76**	0.64	0.90
F	62.72***			11.80***		

*p < 0.05, **p < 0.01, ***p < 0.001

**Table 4:** Logit models predicting whether married women can ask for condom use

	Model 1			Model 2		
	OR	95% CI		OR	95% CI	
**Household structure**						
Monogamous	1.00			1.00		
Polygamous	0.24***	0.19	0.31	0.64***	0.53	0.76
**Region of residence**						
East				1.00		
Adamawa				0.74	0.48	1.14
Centre				0.79	0.54	1.13
Douala				0.29***	0.17	0.50
Far-North				0.15***	0.10	0.23
Littoral				0.26***	0.15	0.42
North				0.15***	0.10	0.22
North-West				0.44***	0.28	0.71
West				0.63**	0.45	0.89
South				0.59*	0.38	0.93
South-West				0.19***	0.13	0.29
Yaoundé				0.59*	0.37	0.93
**Place of residence**						
Urban				1.00		
Rural				0.69***	0.55	0.87
**Age of respondents**						
15–19				1.00		
20–24				0.99	0.72	1.36
25–29				0.97	0.72	1.29
30–34				1.06	0.78	1.44
35–39				0.96	0.69	1.33
40–44				0.98	0.72	1.33
45–49				0.78	0.55	1.09
**Religion**						
Christian				1.00		
Muslim				0.40***	0.32	0.49
Traditionalist				0.25***	0.12	0.49
None				0.75	0.50	1.13
Other				1.57	0.62	3.95
**Adequate HIV knowledge**						
Yes				1.00		
No				0.61***	0.53	0.71
**Ever been tested for HIV**						
Yes				1.00		
No				0.73**	0.57	0.92
**Education**						
Higher education				1.00		
Secondary education				0.70	0.48	1.02
Primary education				0.45***	0.29	0.67
No education				0.27***	0.17	0.42
**Household wealth**						
Richest				1.00		
Richer				0.76*	0.60	0.97
Middle				0.80	0.60	1.07
Poorer				0.63**	0.45	0.87
Poorest				0.45***	0.30	0.66
**Employment**						
Employed				1.00		
Unemployed				0.88	0.75	1.04
F	144.99***			35.26***		

*p < 0.05, **p < 0.01, ***p < 0.001

In addition to household structure, a range of demographic, psychosocial, and socioeconomic factors were associated with safe sex negotiation among married women (see Models 2 and 4 of Table 2). For example, we find that married women from Douala, Far-North, Littoral, North, and South-West Regions have lower odds of reporting the ability to refuse sex and ask for condom use compared to married women in the East Region. Rural women are also less likely to be able to ask for condom use than urban women (OR = 0.69, *p* < 0.001). Compared to their Christian counterparts, Muslim women are less likely to report the ability to refuse sex (OR = 0.40, *p* < 0.001) and ask for condom use (OR = 0.25, *p* < 0.001), while traditional women have lower odds of being able to ask for condom use (OR = 0.25, *p* < 0.001). A lack of psychosocial resources, such as inadequate HIV knowledge and non-uptake of HIV testing, are negatively associated with the ability to refuse sex and ask for condom use. Finally, compared to their more educated and richer counterparts, the less educated had lower odds of reporting the ability to refuse sex and ask for condom use, while married women from poorer households were less likely to be able to ask for condoms. Unemployed women were less likely to refuse sex compared to employed women (OR = 0.76, *p* < 0.01).

## Discussion

Our findings indicate that in the study context of Cameroon, several married women are not able to negotiate for safe sex, with about 33% of women indicating they cannot refuse sex while 50% cannot ask for condom use. These low estimates are problematic and constitute a major hindrance to Cameroon’s goal of achieving the SDG 5 target of empowering women to make informed decisions regarding sexual relations, contraceptive use, and sexual/reproductive health. Moreover, the ability of ongoing national programmes geared towards improving contraceptive use and HIV testing, such as the “Opt-Out” and non-medical cadre HIV testing strategy [47], may not achieve their desired target when women cannot negotiate for safe sex with their partners.

The findings further revealed that married women in polygamous marriages are less likely to have the ability to refuse sex and ask for condom use compared to those in monogamous marriages. This finding is consistent with other studies conducted in SSA that highlight that women in polygamous unions have less decision-making power regarding sexual reproductive health [17, 23, 48]. In Nigeria, for instance, studies found that polygamous marriages negatively impacted women’s attitudes toward safe sex negotiation [17, 23], and that the use of contraceptives is lower in polygamous marriages [49]. Furthermore, a study in Tanzania highlighted that practicing safe sex with their partners may be ineffective as women in polygamous marriages are unsure of the sexual risk behaviours of the co-wives [50]. In addition, due to the high level of competition among wives in high-polygyny environments to have their husband’s attention and the perceived resource allocation that comes with it, they are less likely to oppose their husband’s decisions related to sexual practices or engage in safe sex negotiation [23, 36]. In highly patriarchal societies such as Cameroon, married women in certain polygyny contexts are seen as subordinate to their husbands and coupled with societal expectations of marriage, women may be limited in their ability to make independent decisions [51].

We also found that demographic and psychosocial factors were associated with safer sex negotiation. For example, Muslim women are less likely to report the ability to refuse sex and ask for condom use compared to Christian women. This observation is consistent with findings highlighted in other SSA countries, such as Nigeria [17] and Ghana [52], which argue that Muslim women may be less vocal about participating in decision-making processes within the household, due to the traditional perspectives in place, potentially reducing their ability to negotiate for safe sex. In addition, we found that married women with inadequate HIV knowledge and those who had never been tested for HIV are less likely to negotiate for safe sex. These findings are consistent with similar studies of women’s safe sex negotiation in SSA, stating that women with less knowledge of HIV/AIDs were less likely to negotiate for safer sex [28]. These findings may suggest that these psychosocial resources are important for engaging in safe sex practices [53]. Moreover, women with higher socioeconomic status, such as those who are more educated, wealthier, and employed, had higher odds of reporting the ability to engage in safe sex practices. This observation is supported by previous research indicating that higher socioeconomic status can lead to more independence and assertiveness among married women, potentially enabling them to negotiate within marital sexual relations [23, 27, 54–56].

Despite the key revelations from our study, there are a number of limitations. First, causal inferences cannot be made as the study is cross-sectional. Our findings are thus limited to associations and should be interpreted with caution. Additionally, due to the self-reported nature of our variables, possible biases, such as social desirability, recall and report bias, may arise. As a result, we recommend further qualitative studies to understand the various factors involved with safe sex negotiation. Moreover, previous research has shown that knowledge of sexual partners’ HIV serostatus may be a critical determinant of sexual behaviours [57, 58]; however, we were not able to include this variable due to the limitations of the CDHS. Despite these limitations, our study is consistent with the literature and is the first to explore the link between household structure and safe sex negotiation among married women in Cameroon.

Based on our findings, several policies are recommended. Firstly, as safe sex negotiation is associated with education and wealth, we argue that there is a need to implement more programmes targeted toward promoting and encouraging safe sexual practices in marriage [23]. Involving men in the programs is also essential as they play major roles in household decisions regarding safe sex practices [28]. In the context of improving women’s safe sex negotiation capacity, the importance of economic empowerment cannot be overstated. Providing women with life skills and financial training is essential to improving health and well-being outcomes [59, 60]. Utilizing community health workers is also essential as they can provide hands-on, door-to-door education on the importance of safe sex negotiation [52]. Given religion’s role, religious leaders are a group of stakeholders that can be involved in providing education on safe sex negotiation and counselling [61]. Another policy intervention is to develop a family planning educational program that is shared with new couples when their marriages are being solemnized at the official marriage registrations. By making this program mandatory, newlyweds will be able to better understand the importance of safe sex negotiation [17]. This program can also help encourage people in both monogamous and polygamous relations to have open conversations on safe sex negotiation. Furthermore, as those residing in unstable regions have lower levels of safe sex negotiation, we recommend that initiatives be implemented in these regions that prioritize women’s autonomy. Overall, the policies and interventions that are implemented to improve women’s safe sex negotiation in Cameroon need to focus on those in polygamous unions, those with lower socioeconomic status and women that reside in politically unstable regions. Implementing these strategies will represent a positive step forward in Cameroon’s quest to reduce HIV prevalence, mother-to-child transmission, and other health goals, as well as in meeting the SDG 5 targets.

## Data Availability

The datasets analyzed during the current study are publicly available in the Demographic and Health Survey repository [https://dhsprogram.com/Countries/Country-Main.cfm?ctry_id=4&c=Cameroon].

## Abbreviations

CDHS: Cameroon demographic and health survey
FGM/C: Female genital mutilation or cutting
HIV: Human immunodeficiency virus
ORs: Odds ratios
SSA: Sub-Saharan Africa
SDG: Sustainable development goal
